# Hibernoma: case report of a rare lipomatous tumor^☆^^☆☆^

**DOI:** 10.1016/j.abd.2019.09.018

**Published:** 2019-09-30

**Authors:** Margarida Moura Valejo Coelho, Alexandre João, Cândida Fernandes

**Affiliations:** Department of Dermatology and Venereology, Centro Hospitalar Universitário de Lisboa Central, Lisbon, Portugal

Dear Editor,

We report the case of a 24-year-old female, Fitzpatrick phototype V, referred to our Dermatology Department for an asymptomatic mass in her left dorsal region. The patient reported a slow growth of this mass over several years. Physical examination revealed a palpable, soft, subcutaneous tumor in the left dorsal region, without apparent involvement of the superjacent skin, which was painless on palpation. The remainder of the examination was otherwise normal.

A high-resolution thoracic computed tomography performed one year before, in the context of an episode of asthma exacerbation, had revealed a large, low-density, subcutaneous nodularity in the referred topography (Fig. 1A). Also, an ultrasound-guided core needle biopsy (Fig. 1B)of this well-defined, slightly hyperechoic, subcutaneous mass identified a neoplasm of globular cells, some with multi-vacuolated cytoplasm and others with granular, eosinophilic cytoplasm, without nuclear atypia.

**Figure 1:**
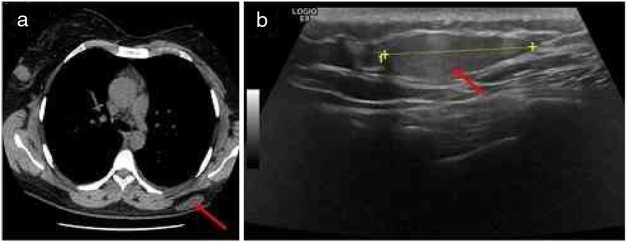
Imagiological features of the tumor: A, High-resolution thoracic computed tomography revealing a large, low-density, subcutaneous nodule in the left dorsal region (red arrow); B, Ultrasound revealing a well-defined, slightly hyperechoic, subcutaneous mass in the left dorsal region (red arrow).

Considering this, we performed a complete surgical tumor resection, under local anesthesia, in an uneventful procedure (Fig. 2A). The tumor measured approximately 60 × 50 × 20 mm, had a gelatinous external surface and, on section, showed a soft consistency and a brownish coloration (Fig. 2A and B). The histopathological examination revealed a hypodermic tumor, involved by a thin fibrous capsule, constituted by adipocytes with granular, eosinophilic cytoplasm, without cytologic atypia, numerous multi-vacuolated adipocytes and some uni-vacuolated cells, establishing the definite diagnosis of a hibernoma (Fig. 3). The patient recovered fully after surgery, without tumor recurrence after six months of follow-up.

**Figure 2:**
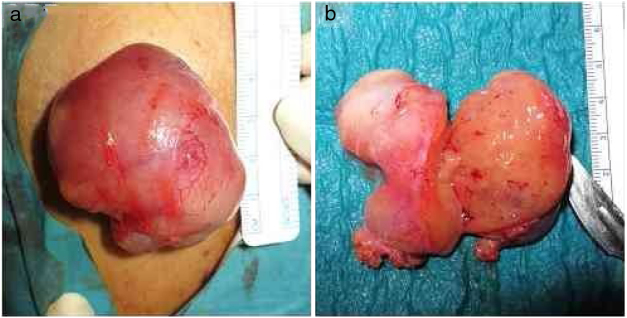
Macroscopic features of the tumor: A, During surgical tumor resection, under local anesthesia; B, After complete excision, the tumor measured approximately 60 × 50 × 20 mm, had a gelatinous external surface and, on section, showed a soft consistency and a brownish coloration.

**Figure 3:**
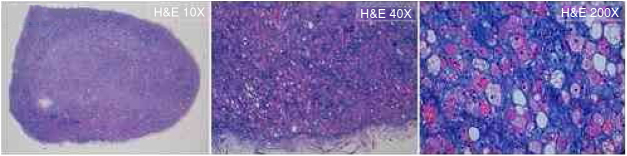
Microscopic features of the tumor: The histopathological examination (hematoxylin & eosin: 10×, 40×, 200×) of the surgical specimen revealed a hypodermic tumor, involved by a thin fibrous capsule, constituted by adipocytes with granular, eosinophilic cytoplasm, without cytologic atypia, numerous multi-vacuolated adipocytes, and some uni-vacuolated cells, establishing the definitive diagnosis of hibernoma.

Hibernomas are rare, benign soft-tissue tumors arising from vestigial brown fat, which can be located in the subcutaneous tissue, the skeletal muscle, or the intermuscular fascia.^1^, ^2^ There are four histological variants of hibernoma: typical (82%), myxoid (9%), lipoma-like (7%), and spindle-cell (2%).^1^ Hibernomas vary in size (1–24 cm, average dimension 9.3 cm) and location, occurring most commonly in the thigh, peri- and interscapular region, neck, arm, abdominal cavity, and retroperitoneum, and they are typically highly vascularized.^1^, ^3^, ^4^, ^5^ They are most often diagnosed in adults (mean age 38 years).^1^

These lipomatous tumors generally present either as slow-growing, painless, soft, palpable and mobile masses, or as incidentalomas in imaging studies.^1^, ^3^, ^4^, ^5^ Symptoms secondary to compression of adjacent structures can also develop due to their growth.^1^, ^3^, ^4^ Differential diagnosis is not always straightforward, and includes not only benign soft-tissue neoplasms (like atypical lipomas, hemangiomas, and angiolipomas) but also malignant, aggressive tumors (namely well-differentiated liposarcomas, myxoid liposarcomas, and rhabdomyosarcomas).^1^, ^2^ In fact, hibernomas can mimic these other tumors clinically, imagiologically, and even histologically, considering some similar features in biopsy specimens.^1^, ^2^, ^3^, ^4^, ^5^

Histopathological examination of the tumor following complete surgical excision, which is curative, is essential for confirming the diagnosis.^1^, ^4^, ^5^

## Author's contribution

Margarida Moura Valejo Coelho: Approval of the final version of the manuscript; elaboration and writing of the manuscript; obtaining, analyzing and interpreting the data; effective participation in research orientation; intellectual participation in propaedeutic and/or therapeutic conduct of the case studied; critical review of the literature; critical review of the manuscript.

Alexandre João: Approval of the final version of the manuscript; obtaining, analyzing and interpreting the data; intellectual participation in propaedeutic and/or therapeutic conduct of the case studied; critical review of the manuscript.

Cândida Fernandes: Approval of the final version of the manuscript; obtaining, analyzing and interpreting the data; intellectual participation in propaedeutic and/or therapeutic conduct of the case studied; critical review of the manuscript.

## Financial support

None declared.

## Conflicts of interest

None declared.
